# Assembly and comparative analysis of the multipartite mitochondrial genome of *Curcuma wenyujin* reveals sequence divergence, structural variation, and phylogenetic placement within Commelinales

**DOI:** 10.3389/fpls.2026.1899169

**Published:** 2026-07-23

**Authors:** Zhihao Lin, Zihan Sun, Ruike Fan, Yiwen Wang, Lishang Dai, Wenfei He

**Affiliations:** 1 School of Traditional Chinese Medicine, Wenzhou Medical University, Wenzhou, China; 2 Department of Rheumatology and Immunology, The Affiliated Hospital of Jiaxing University (The First Hospital of Jiaxing), Jiaxing, China; 3 Jiaxing Key Laboratory of Osteoporosis and Bone Metabolism, The Affiliated Hospital of Jiaxing University (The First Hospital of Jiaxing), Jiaxing, China

**Keywords:** *Curcuma wenyujin*, mitochondrial genome, comparative genomics, phylogeny, RNA editing, codon usage bias

## Abstract

*Curcuma wenyujin*, a perennial herb known for its ornamental and medicinal properties, is a traditional Chinese medicine used for treating rheumatic immune diseases, yet its mitochondrial genome has yet to be studied. This research gap substantially restricts the comprehensive exploration of its genomic resources and phylogenetic relationships. To fill this gap, we assembled and annotated the mitochondrial genome of *C. wenyujin* using a combination of second- and third-generation sequencing technologies. Our results show that the mitochondrial genome is predominantly composed of 26 linear DNA molecules and one circular DNA molecule, with a total length of 7,856,194 bp and a GC content of 43.91%. We identified 125 genes, 90,107 simple repeats, 6,629 tandem repeats, 81,307 dispersed repeats, 2,171 SSRs, and 506 RNA editing sites in the genome. Most protein-coding genes initiate with the canonical ATG start codon, and a strong preference for A or T (U) at the third codon position was observed. The codons UUU, AUU, and UUC are the most frequently used in the mitochondria of *C. wenyujin*. Codon usage bias analysis revealed a strong preference for A/T-ending codons (87.1% of preferred codons), consistent with the conserved pattern observed in angiosperm mitochondrial genomes. Furthermore, phylogenetic analysis was performed based on 24 representative plant species, revealing a close genetic relationship between *C. wenyujin* and *Pontederia crassipes* within the order Commelinales. Comparative analyses with the congener *Curcuma amarissima* (Zingiberaceae) further supported high evolutionary conservation of mitochondrial genomes in the ginger family. This study represents the first comprehensive report on the mitochondrial genome of *C. wenyujin*, laying a solid foundation for future studies on its crop genetics and molecular breeding.

## 1 Introduction

*Curcuma wenyujin* is a perennial herbaceous species belonging to the genus Curcuma (Zingiberaceae). It is widely distributed in subtropical and tropical regions, with its primary production area in Wenzhou, Zhejiang Province, China, where it is ranked first among the ‘Zhejiang Eight Flavors’ authentic medicinal materials. C. wenyujin serves as the source plant for three widely used traditional Chinese medicines (Wei et al., 2022). According to the 2020 edition of the Pharmacopoeia of the People’ s Republic of China, these three medicinal materials derived from the same plant are: “Wen Yujin” (from steamed tubers); “Wen Ezhu” (from steamed rhizomes); and “Pian Jiang Huang” (from freshly sliced rhizomes) (Li et al., 2021; Wei et al., 2022). Current research on *C. wenyujin* has mainly focused on its active components and pharmacological activities. Studies have reported that *C. wenyujin* exhibits anti-inflammatory, antioxidant, anti-arthritic, anti-tumor, antibacterial, and antiviral activities (Yin et al., 2013; Liu et al., 2019; Zhai et al., 2019). To date, more than 150 bioactive compounds have been identified in *C. wenyujin*, including terpenoids (monoterpenes, sesquiterpenes, and diterpenoids), curcumin, polysaccharides, and other active constituents (Chen et al., 2022).

The mitochondrial genome (mtDNA) is a semi-autonomous genetic system in eukaryotes, often referred to as the chondriome. As key organelles (van Loo et al., 2002; Song et al., 2023; Wang et al., 2023; Lim, 2024), mitochondria play pivotal roles in core biological processes, including cellular respiration, energy metabolism, DNA replication and transcription, and tRNA synthesis (Favre et al., 2010; Fu et al., 2019). Mitochondria arose from endosymbiotic prokaryotes and retain independent genetic material and expression systems, despite a dramatic reduction in genome size compared with the nuclear genome (Tanifuji et al., 2017; Dhir et al., 2018). While plant and animal mitochondrial genomes share core functions, plant mitochondrial genomes display remarkable structural divergence, characterized by extensive interspecific variation in genome size, abundant repetitive elements, and frequent structural rearrangements (Liu et al., 2011). This diversity is mainly driven by two mechanisms: (1) repetitive sequences originating from horizontal gene transfer (HGT) of chloroplast or nuclear genome fragments and intronic elements (Mackiewicz et al., 2013; Hedenäs et al., 2021); (2) RNA editing involving nucleotide substitutions, insertions, or deletions. Notably, HGT-mediated DNA fragment integration acts as a major evolutionary force in plants, whereas simple sequence repeats (SSRs) serve as important molecular markers for species identification and genetic diversity evaluation (Mehmood et al., 2020; Wang et al., 2021). RNA editing, which mainly causes non-synonymous mutations at the first and second codon positions, strongly influences gene regulation and may introduce biases in phylogenetic reconstruction (Goremykin et al., 2009; Tseng et al., 2013; Roma et al., 2018). Although the evolutionary conservation of mitochondrial protein-coding genes makes them suitable for high-level phylogenetic analyses, the highly dynamic structural rearrangements (especially in angiosperms) and complex conformations (Mikhailov et al., 2019; Mi et al., 2024) present substantial challenges for genome sequencing and assembly (Chu et al., 2024; Guo et al., 2024). Therefore, elucidating the structural organization and functional regulation of plant mitochondrial genomes is critical for understanding the mechanisms underlying adaptive evolution, interorganellar genetic interactions, and phylogenetic relationships across plant lineages (Howe and Barbrook, 2024).

In recent years, the rapid advancement of genomics and molecular biology technologies has greatly facilitated the dissection of plant genetic backgrounds, the improvement of agronomic traits, and the enhancement of stress resistance (Sudheer et al., 2020). Accordingly, an increasing number of researchers have focused on plant genomic studies. In particular, mitochondrial genomics has attracted growing attention owing to its unique genetic features and essential roles in plant growth and development. To date, only the chloroplast genome of *C. wenyujin* within the Zingiberaceae family has been published (Kim et al., 2021), while mitochondrial genomes have rarely been reported. Recently, the first complete mitochondrial genome of *C. amarissima* (Zingiberaceae) was released, providing a valuable reference for comparative analysis. Therefore, characterizing the mitochondrial genome of *C. wenyujin* will substantially improve our understanding of mitochondrial genome evolution in Zingiberaceae, help identify key genetic factors in this family, and enable the development of mitochondrial DNA barcodes for the authentication of medicinal materials (Li et al., 2019). Although current research primarily focuses on genomic characteristics and phylogenetic relationships (Zheng et al., 2015; Huang et al., 2022; Ma et al., 2025), similar studies in other plant species have successfully identified mitochondrial genes associated with male sterility (Xiao et al., 2022; Liu et al., 2023; Lian et al., 2024; Li et al., 2025). In this study, we present the first comprehensive assembly and annotation of the mitochondrial genome of *C. wenyujin*. We performed comprehensive analyses of gene content, repetitive sequences, RNA editing, codon usage bias, and phylogenetic relationships. These findings significantly improve our understanding of the structural and functional characteristics of the *C. wenyujin* mitochondrial genome. Furthermore, our results provide valuable molecular markers that will support future research on the conservation biology, population genetics, breeding, and evolutionary history of this species.

## 2 Materials and methods

### 2.1 Plant materials collection, DNA extraction, and sequencing

*C. wenyujin* tubers were obtained from the *C. wenyujin* planting base in Ruian. After harvesting, the tubers were processed: roots were removed using high-temperature sterilized scissors, and the tubers were stored in sand until the following spring, then transplanted to a medicinal plant garden. After 60 days of cultivation, fresh leaves were collected and used for sequencing. *C. wenyujin* was formally identified by Associate Professor Zhigang Wu. Voucher leaf specimens were deposited in the publicly accessible Herbarium of Traditional Chinese Medicine, Wenzhou Medical University (voucher number: WYJ203). Samples were frozen in liquid nitrogen and stored at -80 °C until use. Total genomic DNA was extracted using a DNA Secure Plant Kit following the manufacturer’ s protocol (TIANGEN, Beijing, China). DNA quality and concentration were evaluated using agarose gel electrophoresis and spectrophotometry (NanoDrop2000, Thermo Fisher Scientific). A combined strategy of second-generation (Illumina NovaSeq 6000) and third-generation (Oxford Nanopore PromethION) sequencing was employed to balance sequencing depth and long-fragment assembly accuracy. Sequencing statistics for long reads and short reads are summarized in **Supplementary Tables 1**, **2**, respectively. For second-generation sequencing library preparation: DNA was fragmented by ultrasonication, followed by fragment purification, end repair, 3’ A-tailing, adapter ligation, and PCR amplification. The constructed libraries were sequenced on the Illumina Novaseq 6000 platform. Raw reads were filtered using fastp (v0.20.0) (Chen et al., 2018) with the following criteria: (1) Reads containing adapter or primer sequences; (2) Reads with an average Phred quality score < Q5; (3) Reads with an N-base proportion > 5%. For third-generation sequencing and quality control: DNA fragments longer than 10 kb were enriched using magnetic bead-based selection, followed by end repair and adapter ligation using the SQK-LSK109 library preparation kit. Sequencing was performed on the Oxford Nanopore PromethION platform (Korlach et al., 2010). Third-generation sequencing data were filtered using filtlong (v0.2.1) with parameters: --min_length 1000 and --min_mean_q 7. Perl scripts were used to ensure that the data met the requirements for high-accuracy genome assembly.

### 2.2 Mitogenome assembly and annotation

The assembly strategy is as follows: 1). We utilized Minimap 2 (v.2.24) to align the Nanopore reads to our draft assembly of *C. wenyujin* (Li, 2018). 2). The aligned reads were extracted and subjected to *de novo* assembly. 3). Initially, the original third-generation sequencing data were aligned to the seed sequence using minimap 2, screened for sequences with overlap greater than 1 kb, added to the seed sequence, and iteratively aligned the original data to the seed sequence, thus obtaining all the third-generation sequencing data of the mitochondrial genome, and subsequently the resulting three-generation data were corrected using canu (Koren et al., 2017). 4). After that, using Bowtie 2 (v2.3.5.1) (Langmead and Salzberg, 2012) to align the short-reads to the previous correction results, using Unicycler (v0.4.8) (Wick et al., 2017) for mixed assembly, and 5). Split the GFA file according to the coverage of the long reads to obtain the final assembly result.

The mitochondrial genomes were annotated by BLASTN (Alverson et al., 2010). Mitochondrial genes were identified and queried against the NCBI database. Additionally, tRNA genes were detected using tRNAscan-SE software (Chan and Lowe, 2019) (http://lowelab.ucsc.edu/tRNAscan-SE/); NCBI Open Reading Frame (ORF) Finder (http://www.ncbi.nlm.nih.gov/gorf/gorf.html) software was used to annotate the ORF and annotate the nr libraries over three hundred; RNA editing sites were predicted using the PmtREP (http://112.86.217.82:9919/#/tool/alltool/detail/336) software. The boundaries of the introns were manually reviewed and corrected to ensure the complete structure of the protein-coding genes.Mitochondrial genome maps were constructed using the OGDRAW (https://chlorobox.mpimp-golm.mpg.de/OGDraw.html) (Greiner et al., 2019).

### 2.3 Analysis of repeat sequences

Three types of repeat sequences (simple repeat sequences, tandem repeat sequences, and dispersed repeat sequences) were identified in *C. wenyujin*. Simple sequence repeats (SSRs) were detected using MISA software (v1.0) with parameters set to 1-10, 2-5, 3-4, 4-3, 5-3, 6-3. Tandem repeats were identified using trf software (trf409.linux6) with the following settings: 2, 7, 7, 80, 10, 50, 2000, -f, -d, -m. to identify tandem. For dispersed repeats, BLASTN (v2.10.1) was employed with a word size of 7 and an e-value of 1e- 5, ensuring that redundancy and tandem repeats were excluded. The final results were visualized using Circos (v0.69-5).

### 2.4 RSCU analysis

Due to the degeneracy of codons, each amino acid corresponds to a minimum of one codon and a maximum of six codons. There are great differences in the utilization of genome codons in different species and different organisms. This inequality in the use of Synonymous Codon is called Relative Synonymous Codon Usage (RSCU). This preference is thought to be the result of a combination of natural selection, species mutation and genetic drift. Unique CDS sequences were filtered using an in-house Perl script for subsequent analysis.

### 2.5 RNA editing predicting

The principle of RNA editing site prediction in the mitogenome of *C. wenyujin* is based on multi-sequence alignment. Amino acid sequence of the target sequence is compared with multiple sequences in the database, and then the changes of each site of the target sequence in the corresponding position in the database are counted. If the conditions for RNA editing are met, this site may be a potential RNA editing site. So we adopt PmtREP (http://112.86.217.82:9919/#/tool/alltool/detail/336) to predict RNA editing sites in the *C. wenyujin* mitogenome.

### 2.6 Phylogenetic analysis

In order to reveal the phylogeny of *C. wenyujin*, 24 angiosperm mitogenomes (**Supplementary Table 9**) were selected from the NCBI Organelle Genome Resource database (https://www.ncbi.nlm.nih.gov/genome/browse/#!/organelles/). Using PhyloSuite (v1.2.2), we extracted the shared protein-coding genes (PCGs) among the mitochondrial genomes of the selected species (Zhang et al., 2020). MAFFT (v7.427) was utilized for aligning the extracted sequences (Katoh and Standley, 2013). The maximum likelihood (ML) method implemented in RAxML v.8.2.10. The parameters were ‘raxmlHPC-PTHREADS-SSE3 -f a -N 1000 -m GTRGAMMA -x 551314260 -p 551,314,260’. The bootstrap analysis was performed with 1,000 replicates. Bayesian inferences (BI) analysis was performed by MrBayes (v3.2.7) with the GTR + G + I model and 1000 bootstrap replicates.

## 3 Results

### 3.1 Mitogenome assembly and annotation results

**Figure 1:**
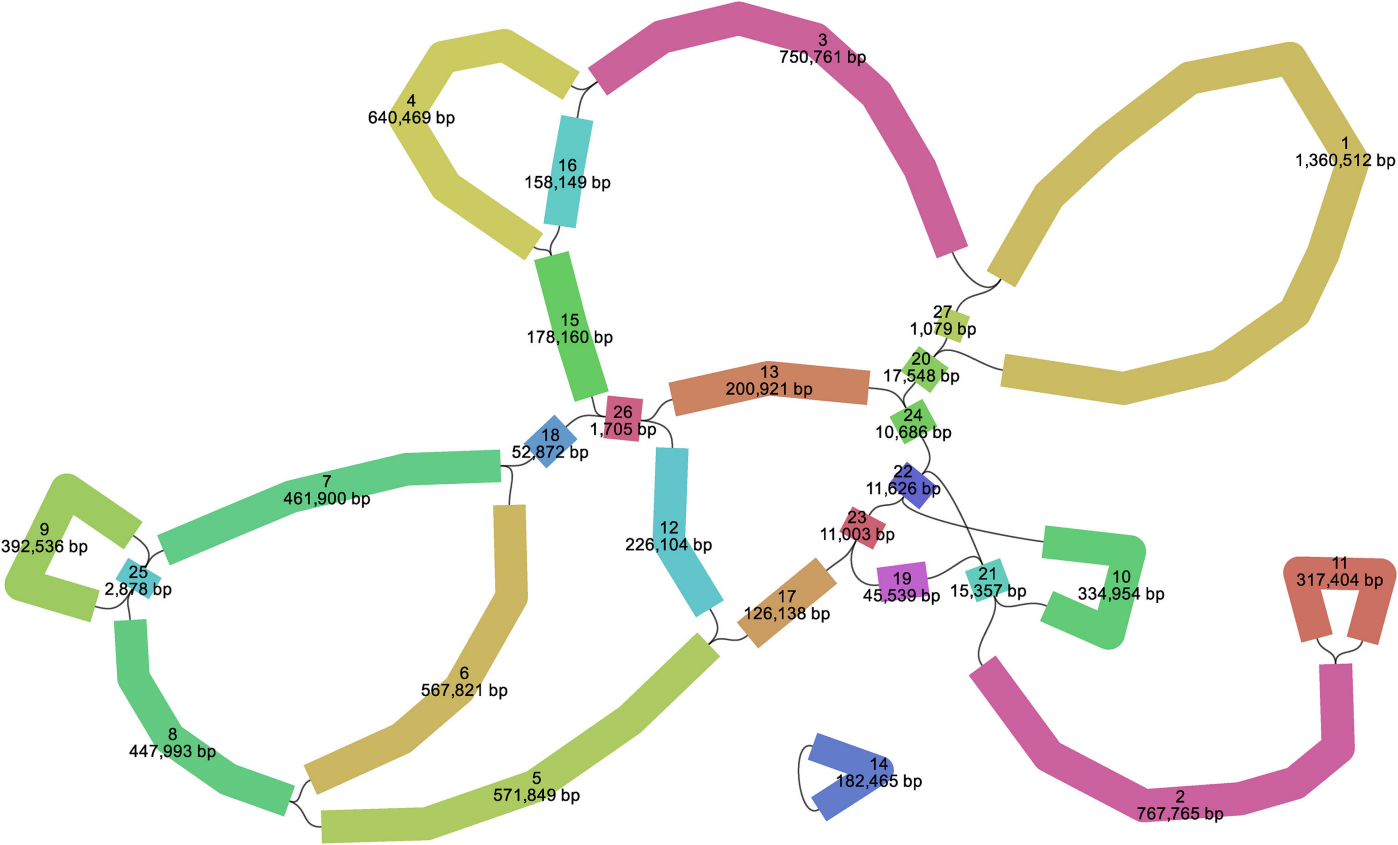
Mitochondrial genome assembly topology in GFA (Graphical fragment assembly) format. Bandage visualization revealed that the mitochondrial contigs of *C. wenyujin* exhibit complex multi-circular architecture (including master and subgenomic circles), indicative of dynamic recombination events and structural heteroplasmy characteristic of plant mitochondrial genomes.

**Figure 2:**
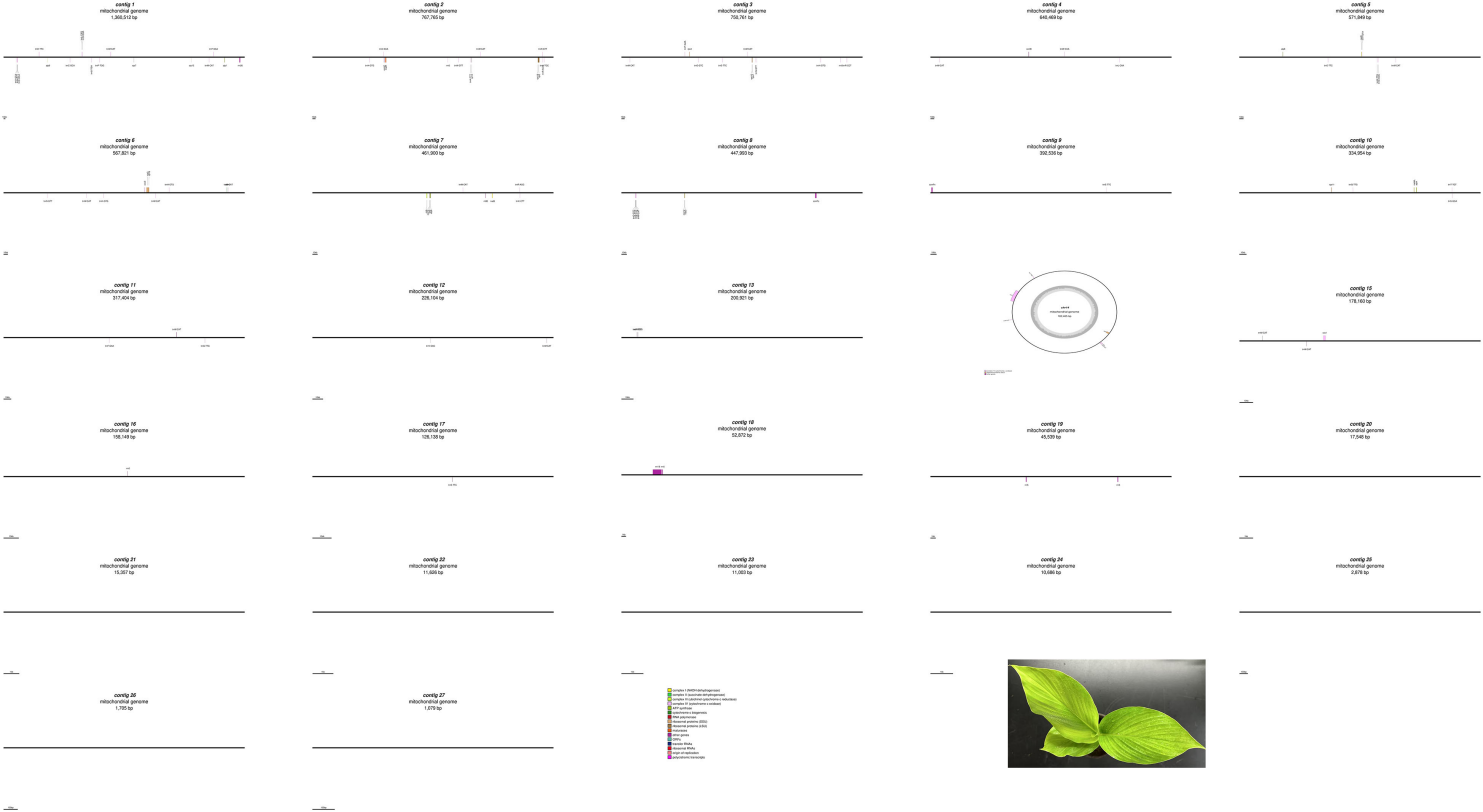
The mitochondrial genome annotations for *C. wenyujin*. Sequence features are labeled on each circle. Different colors correspond to different roles of genes.

**Figure 3:**
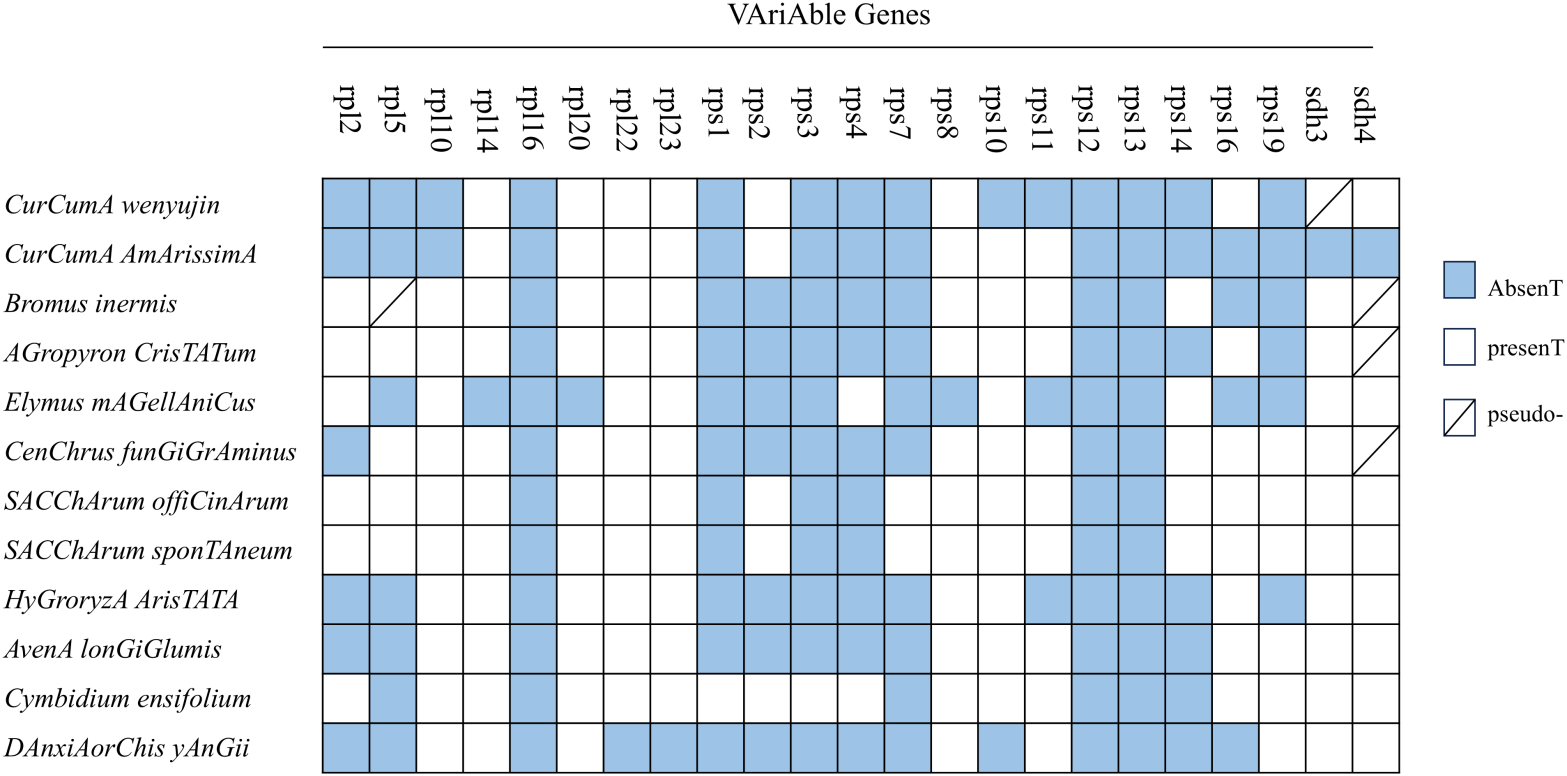
Mitochondrial gene composition across 12 plant species. Blue indicates existence; White indicates missing; Slash represents pseudogenes.

Through the utilization of second- and third-generation sequencing technologies, the mitochondrial genome data of *C. wenyujin* were obtained, with an average read length of 10,087 bp (**Supplementary Tables 1**, **2**). The mitogenome was assembled into 27 molecules (as shown in **Figures 1**, **2**), of which only one circular molecule with a length of 182,465 bp (labeled 14), and the rest were linear molecules. The total length is 7,856,194 bp, the shortest length is 1,705 bp (WYJ-27), and the maximum length can reach 1,360,512 bp (WYJ-1). The GC content was 43.91%, similar to the typical values for angiosperms, and the GC content in the single structure of *C. wenyujin* ranged from 38.77% to 45.72% (**Supplementary Table 3**). At the same time, a total of 125 unique genes were identified, including 41 protein-coding genes (PCGs), 66 tRNAs, 9 rRNAs, and 9 pseudogenes (**Table 1**). The total length of protein-coding genes was 35,928 bp, accounting for 0.46% of the total length. Of the PCGs, 26 were classified as core, while 15 were categorized as non-core. 26 core genes included 5 ATP synthase genes (*atp1, atp4, atp6, atp8, atp9*), 4 cytochrome C biogenetic genes (*ccmB, ccmC, ccmFc, ccmFn*), 3 cytochrome C oxidase genes (*cox1, cox2, cox3*), 1 panthenol cytochrome c reduc ubiquinol cytochrome c reductase gene tase gene (*cob*) and 1 maturase gene (*matR*), 11 NADH dehydrogenase genes (*nad1* (x3), *nad2, nad3, nad4, nad4L, nad5, nad6, nad7, nad9*) and 1 Transport membrane protein (*mttB*). This gene composition is highly consistent with the conserved genes of plant mitochondria. The non-core genes comprise 4 ribosomal large subunit genes (*rpl10, rpl16, rpl2 and rpl5*), 11 small subunits of the ribosome (*rps1, rps10, rps11, rps12, rps13, rps14, rps19, rps3, rps4* (x2) and *rps7*). The distribution of these genes varies significantly across plant families and genera, frequently involving gene loss or pseudogenization. However, in *C. wenyujin*, these genes retain intact functionality. Combined with genomic features of the Zingiberaceae species *C. amarissima*, our study demonstrates that mitochondrial genomes in Zingiberaceae exhibit a high degree of evolutionary conservation (**Figure 3**). The total length of 66 tRNA genes is 4,850 bp (0.06%), comprising the following: *trnA-TGC, trnC-GCA* (x3)*, trnD-GTC* (x2)*, trnE-CTC, trnE-TTC* (x6)*, trnF-GAA* (x3)*, trnG-GCC, trnH-GTG* (x4)*, trnK-CTT, trnK-TTT, trnL-CAA, trnL-CAG, trnL-TAG, trnM-CAT* (x16)*, trnM-CAT, trnN-GTT* (x5)*, trnP-TGG* (x2)*, trnQ-TTG* (x3)*, trnR-ACG* (x2)*, trnR-CCT, trnS-GCT, trnS-GCT, trnS-GGA* (x2)*, trnS-TGA, trnT-TGT, trnV-GAC, trnW-CCA*, and *trnY-GTA* (x2). The total length of 9 rRNA genes is 7,061 bp (0.09%), including *rrn18, rrn26*, and *rrn5* (x7). and 9 pseudogenes, namely *atp9, ccmB, cox1, cox2, nad3, nad9, rps1* and *sdh3* (x2), respectively. A total of 13 genes containing introns were identified. *nad1, nad2, nad5, nad7* with 4 introns, *nad4* with 3 introns, *cox2* with 2 introns, and *ccmFc, rpl10, rpl2, rps10, rps3, trnA-TGC, trnI-CAG, trnN-GTT* with 1 intron have 15 genes with multiple copies. Two copies of *rps4, sdh3, trnD-GTC, trnP-TGG, trnR-ACG, trnS-GGA, trnY-GTA*, 3 copies of *trnF-GAA, trnQ-TTG, trnC-GCA*, 4 copies of trnF-GAA*, trnH-GTG*, 5 copies of *trnN-GTT*, 6 copies of *trnE-TTC*, 7 copies of *rrn5*, and 16 copies of *trnM-CAT*. The core gene set was highly consistent with that of *C. amarissima*, supporting high evolutionary conservation in Zingiberaceae (**Figure 3**).

**Table 1:** Genes predicted in the mitogenome of *C. wenyujin*.

Group of genes	Gene name
ATP synthase	#atp9 atp1 atp4 atp6 atp8 atp9
Cytohrome c biogenesis	#ccmB ccmB ccmC ccmFc* ccmFn
Ubichinol cytochrome c reductase	cob
Cytochrome c oxidase	#cox1 #cox2 cox1 cox2** cox3
Maturases	matR
Transport membrance protein	mttB
NADH dehydrogenase	#nad3 #nad9 nad1**** nad2**** nad3 nad4*** nad4L nad5**** nad6 nad7**** nad9
Ribosomal proteins (LSU)	rpl10* rpl16 rpl2* rpl5
Ribosomal proteins (SSU)	#rps1 rps1 rps10* rps11 rps12 rps13 rps14 rps19 rps3* rps4(2) rps7
Succinate dehydrogenase	#sdh3(2)
Ribosomal RNAs	rrn18 rrn26 rrn5(7)
Transfer RNAs	trnA-TGC* trnC-GCA(3) trnD-GTC(2) trnE-CTC trnE-TTC(6) trnF-GAA(3) trnG-GCC trnH-GTG(4) trnK-CTT trnK-TTT trnL-CAA trnL-CAG* trnL-TAG trnM-CAT(16) trnM-CAT* trnN-GTT(5) trnP-TGG(2) trnQ-TTG(3) trnR-ACG(2) trnR-CCT trnS-GCT trnS-GCT* trnS-GGA(2) trnS-TGA trnT-TGT trnV-GAC trnW-CCA trnY-GTA(2)

intron number (* = 1, ** = 2, *** = 3, **** = 4); #Gene, Pseudo gene; Gene(2), Number of copies of multi-copy genes.

### 3.2 Repeat sequence of the mitogenome of C. wenyujin

**Figure 4:**
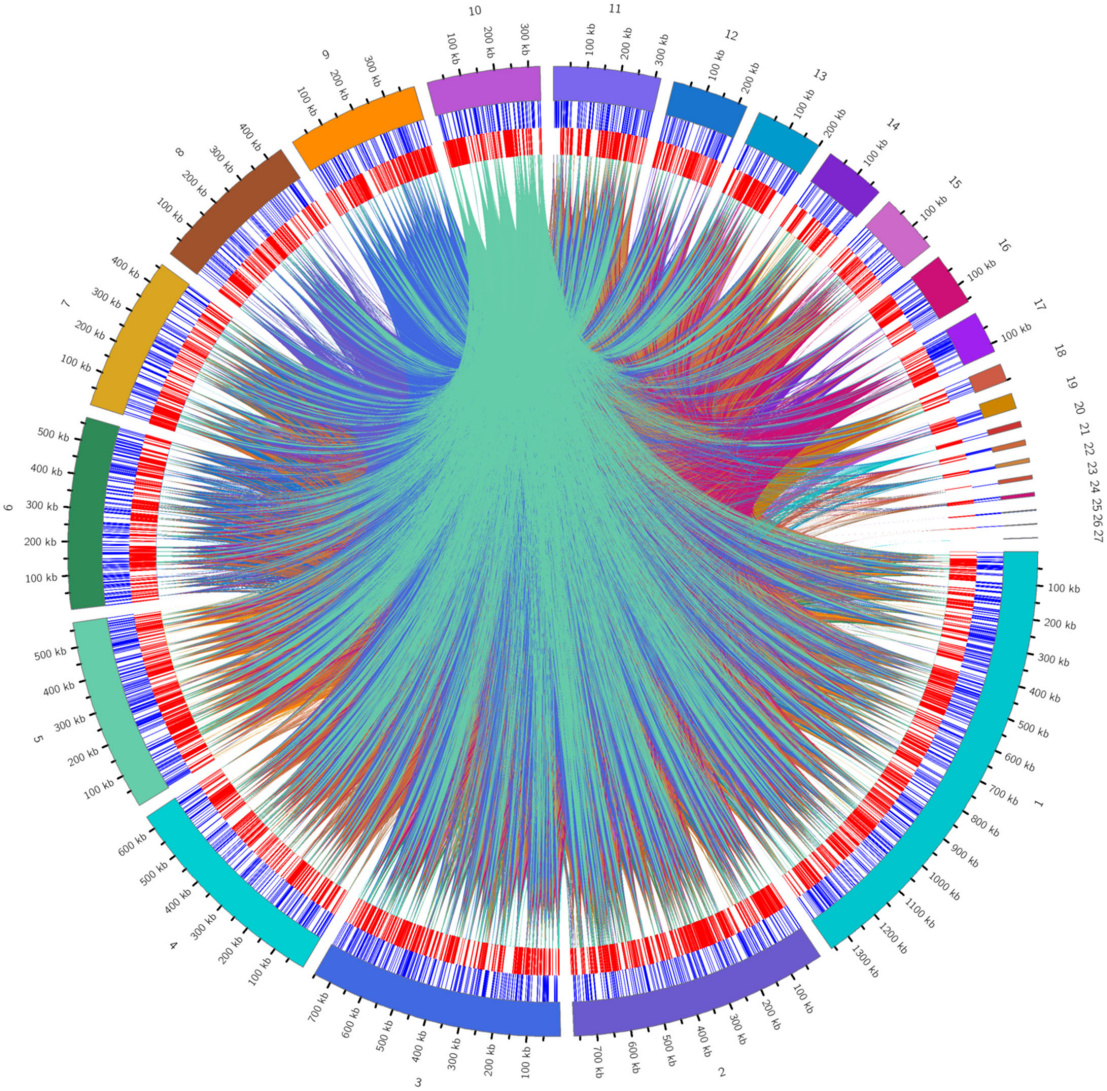
Map of the distribution of repeated sequences across the genome. The outermost ring represents the mitochondrial genome sequence, and the inner sequence is simple repeat sequence, tandem repeat sequence, and scattered repeat sequence.

Simple sequence repeat (SSR) molecular markers are a valuable tool for genetic diversity and population genetic variation analysis due to their high polymorphism and reliability. The repeated sequence of *C. wenyujin* was identified and the results were visualized by circos v0.69–5 software, and the results as shown in **Figure 2** were obtained. It can be seen from the figure that among the 27 subrings of *C. wenyujin*, the length of repeat sequence of component molecule 1 has the largest span, ranging from 100 kb to 1,300 kb, and all three types of repeat sequences exist. Then, with the increase of molecular sequence number, the length of repeat sequence decreases continuously. In the 27th molecule, only scattered repeat sequences exist and the length is less than 100 kb (**Figure 4**).

**Figure 5:**
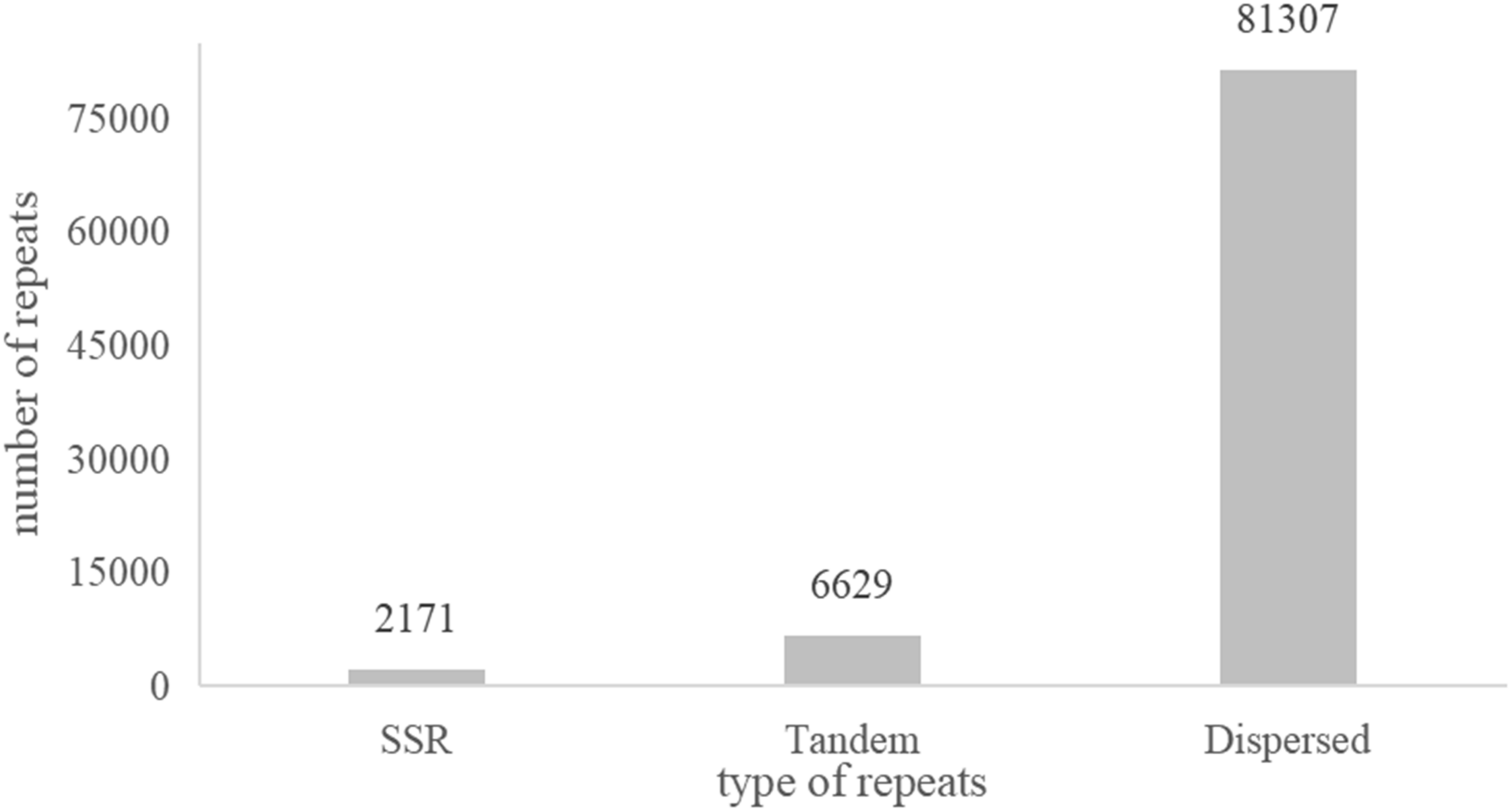
Distribution of repeat sequences. The x-axis represents the type of repeats, while the y-axis represents the number of repeats.

**Figure 6:**
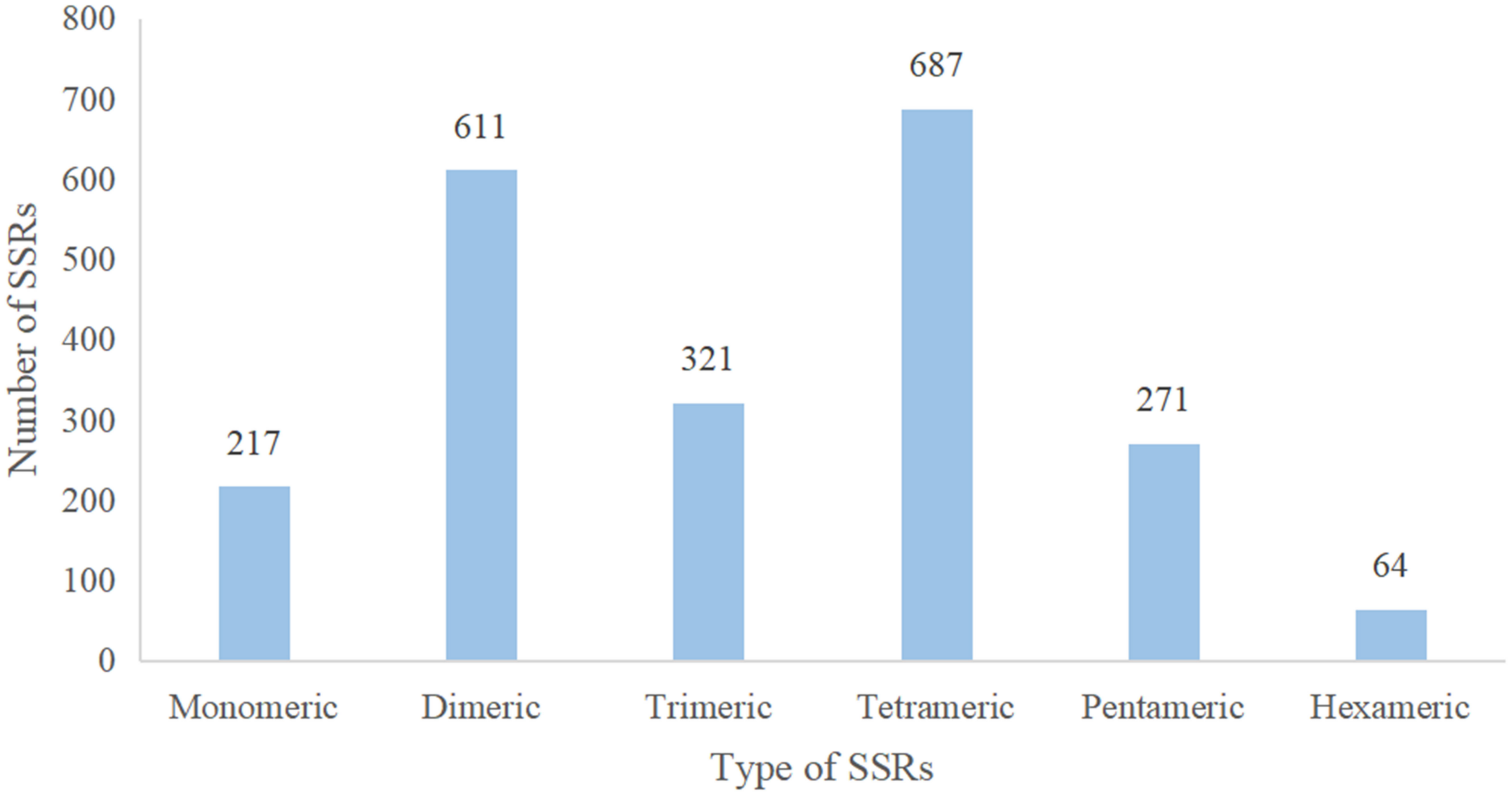
Nucleotide repeat unit of *C. wenyujin* mitogenome. The x-axis represents the type of SSRs while the y-axis represents the number of repeats.

**Figure 7:**
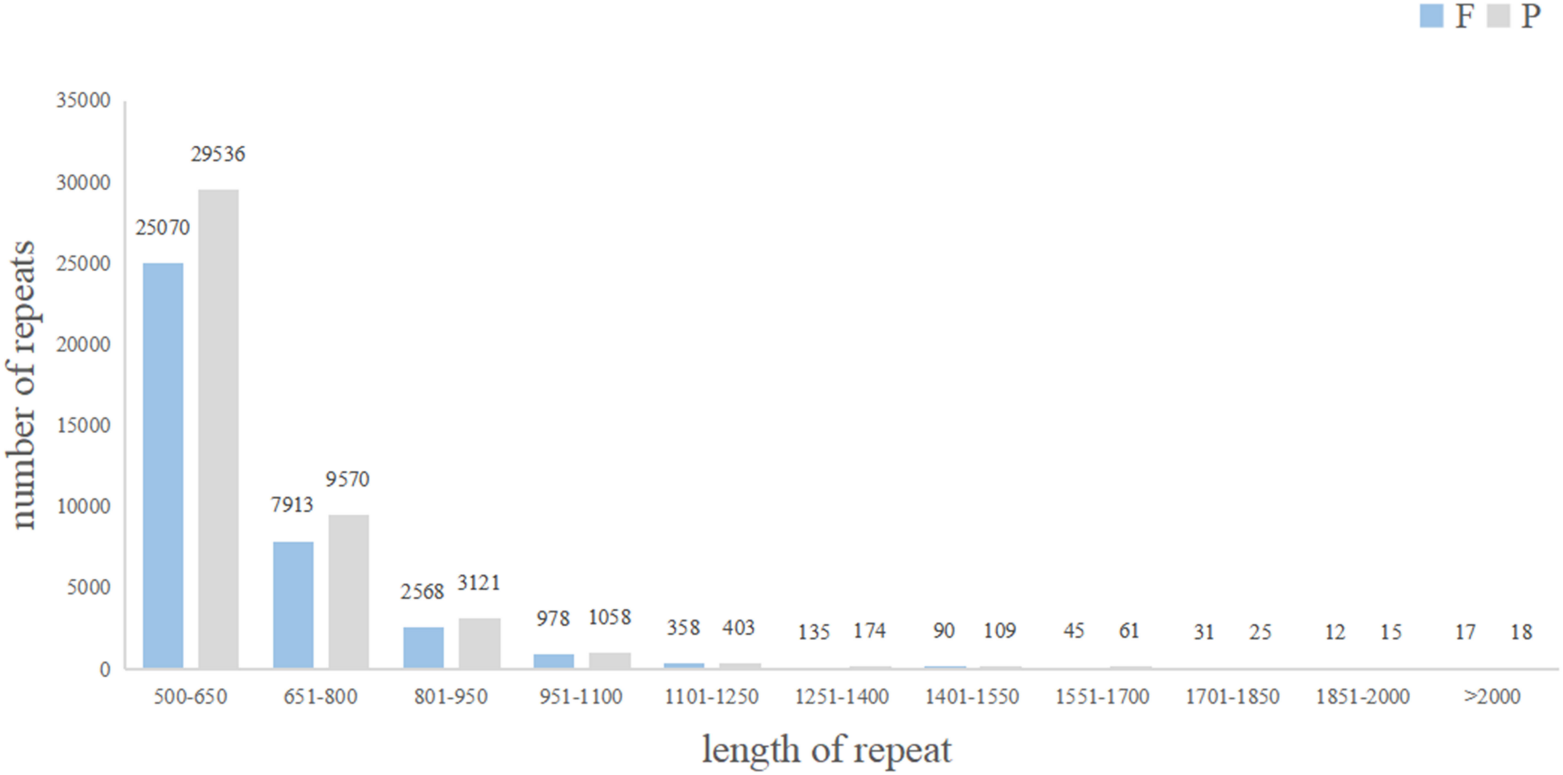
Distributed repeat sequence length allocation. The x-axis displays the length of repeats, and the y-axis displays the number of repeats.

We used MISA software to identify simple repeats, and found a total of 90,107 repeats, including 6,629 tandem repeats, 81,307 scattered repeats, and 2,171 SSR (**Figure 5**). In SSR, tetramers accounted for the largest proportion (31.6%), up to 687, followed by dimers (28.1%) and trimers (14.8%), 611 and 321, respectively, while hexamers accounted for the least, only 64 (**Figure 6**). At the same time, we found that there were 2,127 repeat sequences containing A/T, accounting for 98.0% of the total SSR, while there were only 44 without A/T sequence. Further analysis revealed that 475 of the A/T repeats were composed entirely of A/T. There were 182 monomers (A/T), 192 dimers (AT/AT), 61 trimers (AAT/ATT), 28 tetramers (AAAT/ATTT, AATT/AATT), 11 pentamers (AAAAT/ATTTT, AAATT/AATTT) and 1 hexamer (AAATAT/ATATTT) PC (**Supplementary Table 4**). The A/T richness of 2,127 SSRS ranged from 20% to 100%. There were 44,090 palindromic repeats and 37,217 forward repeats, accounting for 54.2% and 45.8% of the total. Moreover, the length of repeated sequences was not evenly distributed, with 78,805 repeating sequences mostly concentrated between 500-1,000 bp, accounting for 96.9% of the total number, and 2,503 repeating sequences exceeding 1,000 bp, accounting for 3.1% of the total number (**Figure 7**), among which the largest sequence length could reach 2,881 bp. Among these 6,629 tandem repeats, the lengths were distributed from 2 to 1,727 bp, with a match of over 58% and a minimum copy number of 1.8, while the maximum copy number was 48 times, or ‘TA’ (**Supplementary Table 5**). These findings lead us to hypothesize that the expansion of the *C. wenyujin* mitochondrial genome is predominantly driven by repetitive sequences, which may serve as potential molecular markers for the genetic fingerprinting and precise identification of this species. The abundance and distribution of repeats were similar to those of *C. amarissima*, suggesting conserved mechanisms underlying genome expansion in *C. wenyujin*.

### 3.3 Analysis of codon usage in *C. wenyujin*

**Figure 8:**
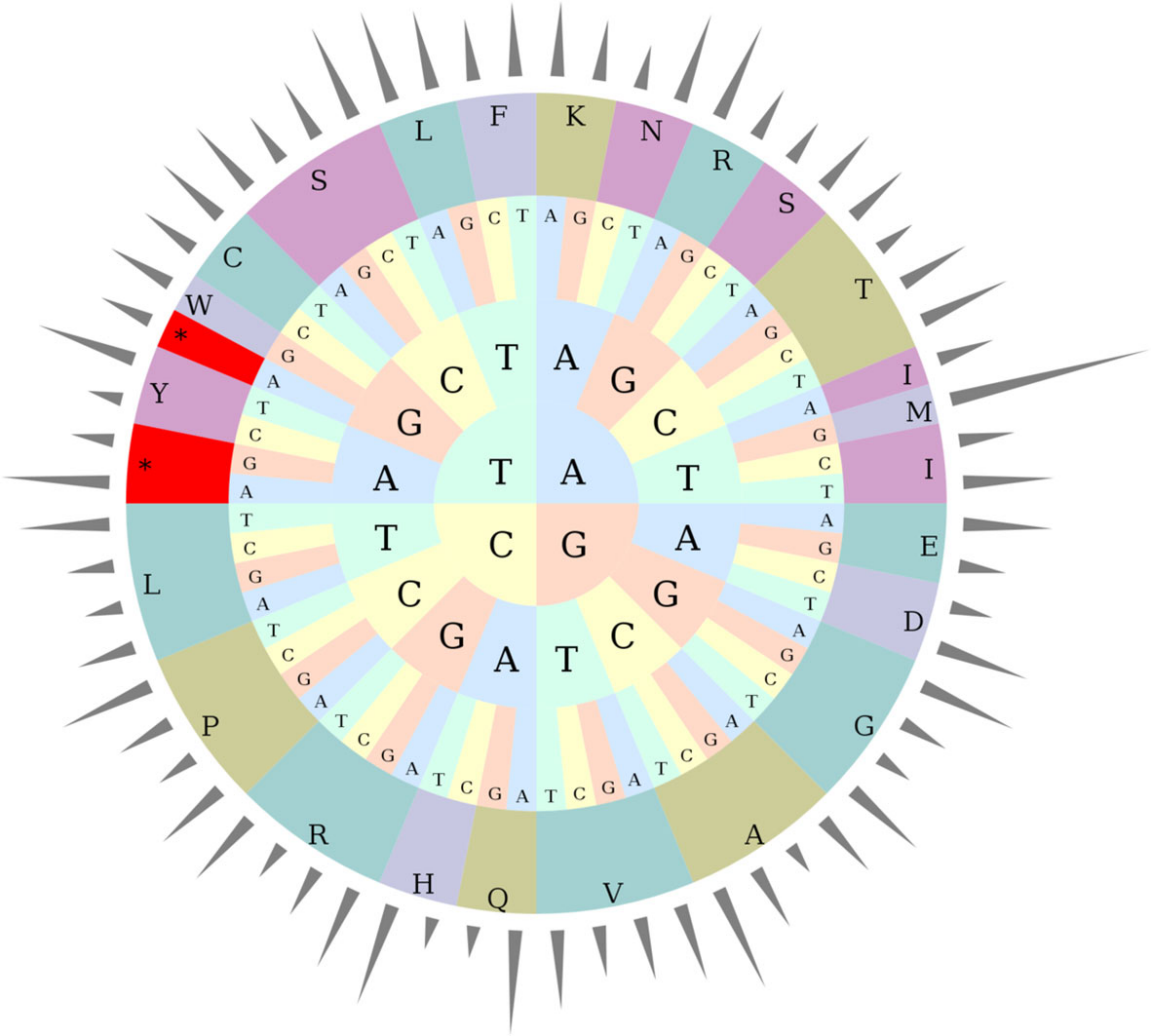
Pie chart of RSCU. The height of the outermost cylinder is the RSCU value, the inner layer is the amino acid, the innermost three layers are the codon.

**Figure 9:**
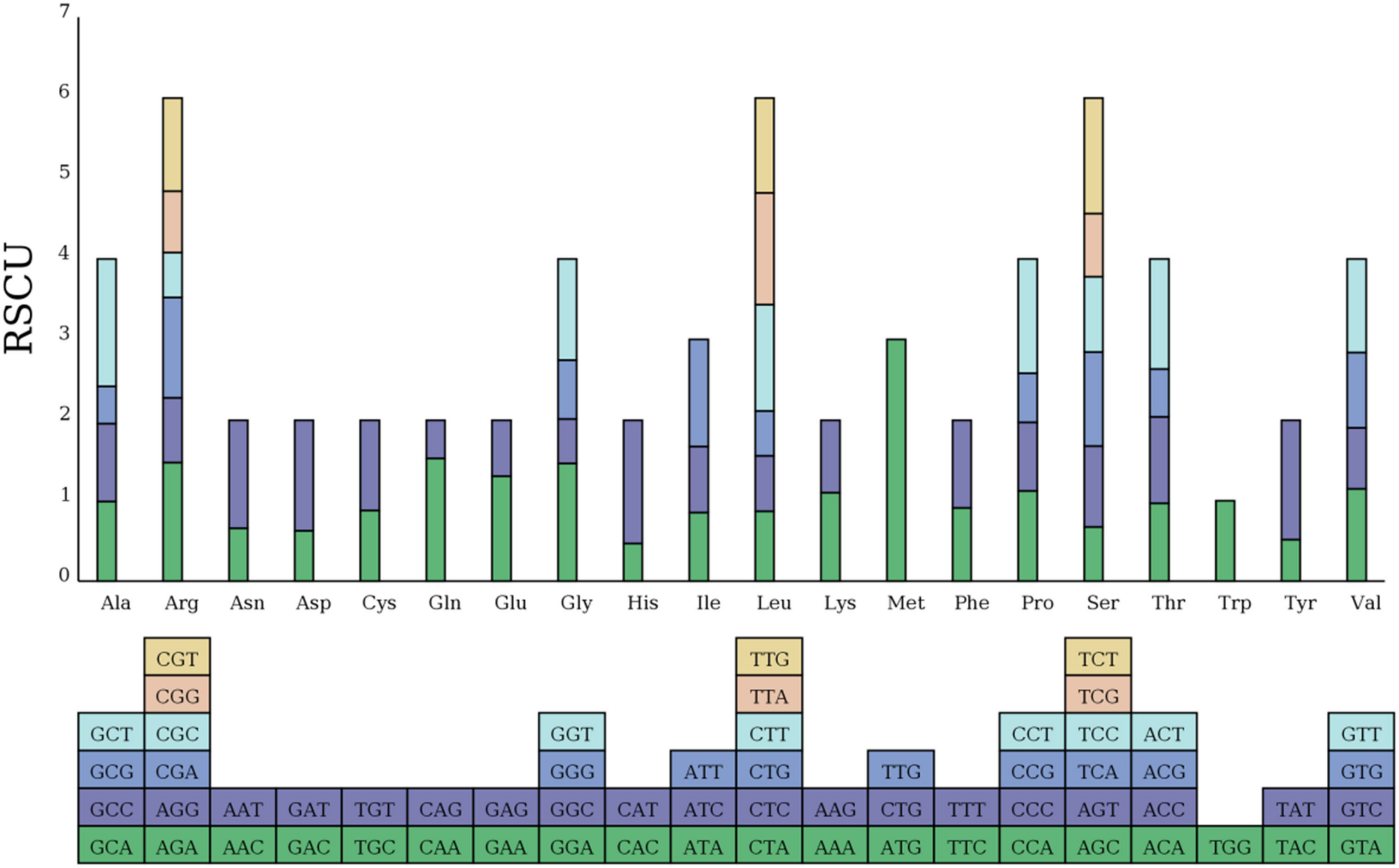
Analysis of codon usage bias in *C. wenyujin* mitogenome. X-axis, codon families; Y-axis, the relative synonymous codon usage (RSCU) value. RSCU measures the likelihood of a specific codon being used among synonymous codons that encode the same amino acid and values greater than 1 indicate a higher frequency of usage for the codon.

In this study, we conducted a comprehensive analysis of codon usage patterns in 41 protein-coding genes (PCGs) of *C. wenyujin*. Our findings revealed that these genes collectively utilize 66 distinct codons (**Table 2**), with a total occurrence frequency of 11,044, encoding more than 20 different proteins. Among the amino acids analyzed, Met and Trp were uniquely encoded by a single codon, which exhibited the highest frequency (**Figures 8**, **9**). In contrast, all other amino acids were encoded by multiple codons, with Leu, Ser, and Arg demonstrating the highest degeneracy, each being encoded by six different codons. These observations are consistent with the general patterns observed in the mitochondrial genomes of angiosperms.

**Table 2:** Mitogenome codon count of *C. wenyujin*.

CODON	COUNT	CODON	COUNT	CODON	COUNT
UAA(*)	20	AUC(I)	226	AGG(R)	104
UAG(*)	8	AUU(I)	363	CGA(R)	162
UGA(*)	10	AAA(K)	275	CGC(R)	72
GCA(A)	176	AAG(K)	225	CGG(R)	99
GCC(A)	171	CUA(L)	172	CGU(R)	150
GCG(A)	82	CUC(L)	137	AGC(S)	114
GCU(A)	281	CUG(L)	111	AGU(S)	170
UGC(C)	73	CUU(L)	262	UCA(S)	197
UGU(C)	93	UUA(L)	275	UCC(S)	158
GAC(D)	109	UUG(L)	234	UCG(S)	132
GAU(D)	238	AUG(M)	300	UCU(S)	243
GAA(E)	300	CUG(M)	0	ACA(T)	135
GAG(E)	159	UUG(M)	0	ACC(T)	149
UUC(F)	314	AAC(N)	112	ACG(T)	83
UUU(F)	375	AAU(N)	228	ACU(T)	190
GGA(G)	286	CCA(P)	168	GUA(V)	192
GGC(G)	107	CCC(P)	127	GUC(V)	126
GGG(G)	143	CCG(P)	92	GUG(V)	156
GGU(G)	245	CCU(P)	212	GUU(V)	194
CAC(H)	64	CAA(Q)	229	UGG(W)	157
CAU(H)	209	CAG(Q)	71	UAC(Y)	81
AUA(I)	233	AGA(R)	191	UAU(Y)	234

The relative synonymous codon usage (RSCU) values greater than 1 indicate a preference for specific codons, reflecting biased usage of certain amino acids, whereas values less than 1 suggest the opposite tendency. In our investigation of the *C. wenyujin* mitochondrial genome, we identified significant codon usage biases that exceeded the standard UGG preference (**Table 2**; **Supplementary Table 6**). Notably, the AUG codon, which exhibited the highest RSCU value of 3.00, was the most frequently occurring codon in the mitochondrial genome. This was followed by GCU (encoding alanine) and UAA (a stop codon), both demonstrating RSCU values of 1.58.

Using an RSCU threshold of 1 as the demarcation point, we observed that the mitochondrial genome displayed pronounced preferences for 31 specific codons. Among these preferred codons, 27 (87.1%) terminated with either A or T (U) bases, comprising 11 A-ending and 16 T-ending codons. This striking predominance of A/T-ending codons (p < 0.01) reveals a substantial A/T bias in codon usage, underscoring a strong evolutionary preference for specific nucleotide terminations in this species.

**Figure 10:**
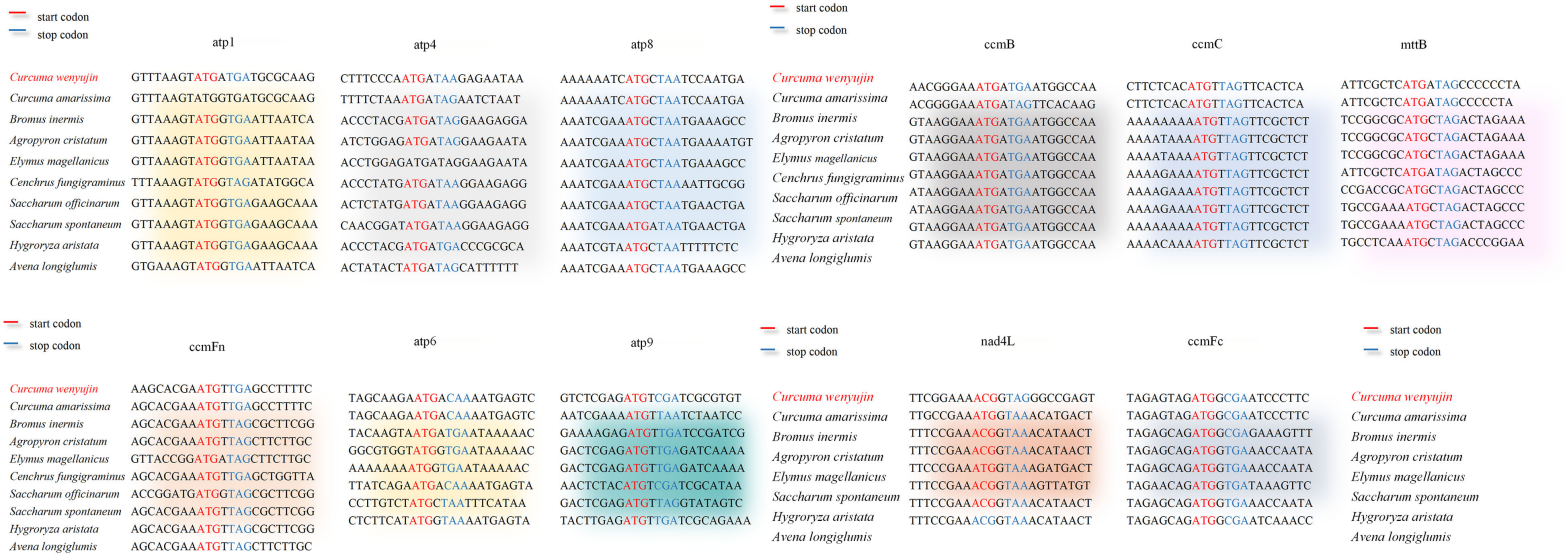
Codon position annotation of plant mitochondrial core genes. Red indicates start codons; blue indicates stop codons.

Most of *C. wenyujin*’ s PCGs have typical ATG start codons and stop codons of TAG, TAA, and TCG, but the start codon of *nad1, nad4L*, and *rps10* is ACG, which is speculated to be the result of C-U RNA editing at the second site. At the same time, the stop codon of *atp6* is CAA, and the stop codon of *atp9* and *ccmFc* is CGA, so C-U RNA editing also occurs in the stop codon (**Table 3**). Furthermore, comparative analysis of *C. wenyujin* with *C. amarissima*, *Bromus inermis*, *Agropyron cristatum*, *Elymus magellanicus*, *Cenchrus fungigraminus*, *Saccharum officinarum*, *Saccharum spontaneum*, *Hygroryza aristata*, and *Avena longiglumis* revealed remarkable conservation of start and stop codons in core genes across these phylogenetically diverse species. Regarding codon-altered core genes, nucleotide substitutions predominantly occurred at the first and second positions of codons (**Figure 10**). Start and stop codons of core genes were highly conserved between C. *wenyujin* and related species.

**Table 3:** Gene profile and organization of the *C. wenyujin* mitogenome.

Group of genes	Gene name	Length	Start codon	Stop codon	Amino acid
ATP synthase	atp1	1524	ATG	TGA	508
	atp4	582	ATG	TAA	194
	atp6	792	ATG	CAA(TAA)	264
	atp8	471	ATG	TAA	157
	atp9	225	ATG	CGA(TGA)	75
Cytohrome c biogenesis	ccmB	609	ATG	TGA	203
	ccmC	723	ATG	TAG	241
	ccmFc	1296	ATG	CGA(TGA)	432
	ccmFn	1710	ATG	TGA	570
Ubichinol cytochrome c reductase	cob	1182	ATG	TGA	394
Cytochrome c oxidase	cox1	1578	ATG	TAA	526
	cox2	768	ATG	TAA	256
	cox3	798	ATG	TGA	266
Maturases	matR	2424	ATG	TAG	808
Transport membrance protein	mttB	348	ATG	TAG	116
NADH dehydrogenase	nad1	978	ACG(ATG)	TAA	326
	nad1	978	ACG(ATG)	TAA	326
	nad1	978	ACG(ATG)	TAA	326
	nad2	1467	ATG	TAA	489
	nad3	357	ATG	TAA	119
	nad4	1488	ATG	TGA	496
	nad4L	294	ACG(ATG)	TAG	98
	nad5	2022	ATG	TAA	674
	nad6	606	ATG	TAA	202
	nad7	1185	ATG	TAG	395
	nad9	573	ATG	TAA	191
Ribosomal proteins (LSU)	rpl10	402	ATG	TAA	134
	rpl16	516	ATG	TAA	172
	rpl2	1533	ATG	TAG	511
	rpl5	552	ATG	TAA	184
Ribosomal proteins (SSU)	rps1	603	ATG	TAA	201
	rps10	417	ACG(ATG)	TAA	139
	rps11	519	ATG	TAA	173
	rps12	378	ATG	TGA	126
	rps13	351	ATG	TGA	117
	rps14	303	ATG	TAG	101
	rps19	270	ATG	TAA	90
	rps3	1680	ATG	TAG	560
	rps4	960	ATG	TAA	320
	rps4	1041	ATG	TAA	347
	rps7	447	ATG	TAA	149
Succinate dehydrogenase					
Ribosomal RNAs	rrn18	1920	–	–	–
	rrn26	4302	–	–	–
	rrn5	121	–	–	–
	rrn5	120	–	–	–
	rrn5	120	–	–	–
	rrn5	119	–	–	–
	rrn5	119	–	–	–
	rrn5	119	–	–	–
	rrn5	121	–	–	–
Transfer RNAs	trnA-TGC	65	–	–	–
	trnC-GCA	71	–	–	–
	trnC-GCA	73	–	–	–
	trnC-GCA	73	–	–	–
	trnD-GTC	74	–	–	–
	trnD-GTC	74	–	–	–
	trnE-CTC	84	–	–	–
	trnE-TTC	72	–	–	–
	trnE-TTC	72	–	–	–
	trnE-TTC	72	–	–	–
	trnE-TTC	72	–	–	–
	trnE-TTC	73	–	–	–
	trnE-TTC	72	–	–	–
	trnF-GAA	73	–	–	–
	trnF-GAA	75	–	–	–
	trnF-GAA	59	–	–	–
	trnG-GCC	72	–	–	–
	trnH-GTG	73	–	–	–
	trnH-GTG	74	–	–	–
	trnH-GTG	74	–	–	–
	trnH-GTG	74	–	–	–
	trnK-CTT	72	–	–	–
	trnK-TTT	73	–	–	–
	trnL-CAA	81	–	–	–
	trnL-CAG	75	–	–	–
	trnL-TAG	80	–	–	–
	trnM-CAT	73	–	–	–
	trnM-CAT	73	–	–	–
	trnM-CAT	69	–	–	–
	trnM-CAT	73	–	–	–
	trnM-CAT	74	–	–	–
	trnM-CAT	73	–	–	–
	trnM-CAT	73	–	–	–
	trnM-CAT	85	–	–	–
	trnM-CAT	73	–	–	–
	trnM-CAT	59	–	–	–
	trnM-CAT	74	–	–	–
	trnM-CAT	72	–	–	–
	trnM-CAT	68	–	–	–
	trnM-CAT	63	–	–	–
	trnM-CAT	77	–	–	–
	trnM-CAT	74	–	–	–
	trnM-CAT	65	–	–	–
	trnN-GTT	72	–	–	–
	trnN-GTT	72	–	–	–
	trnN-GTT	72	–	–	–
	trnN-GTT	78	–	–	–
	trnN-GTT	72	–	–	–
	trnP-TGG	74	–	–	–
	trnP-TGG	75	–	–	–
	trnQ-TTG	71	–	–	–
	trnQ-TTG	72	–	–	–
	trnQ-TTG	72	–	–	–
	trnR-ACG	74	–	–	–
	trnR-ACG	74	–	–	–
	trnR-CCT	68	–	–	–
	trnS-GCT	72	–	–	–
	trnS-GCT	88	–	–	–
	trnS-GGA	87	–	–	–
	trnS-GGA	60	–	–	–
	trnS-TGA	92	–	–	–
	trnT-TGT	73	–	–	–
	trnV-GAC	72	–	–	–
	trnW-CCA	74	–	–	–
	trnY-GTA	83	–	–	–
	trnY-GTA	83	–	–	–

### 3.4 RNA editing analysis

**Figure 11:**
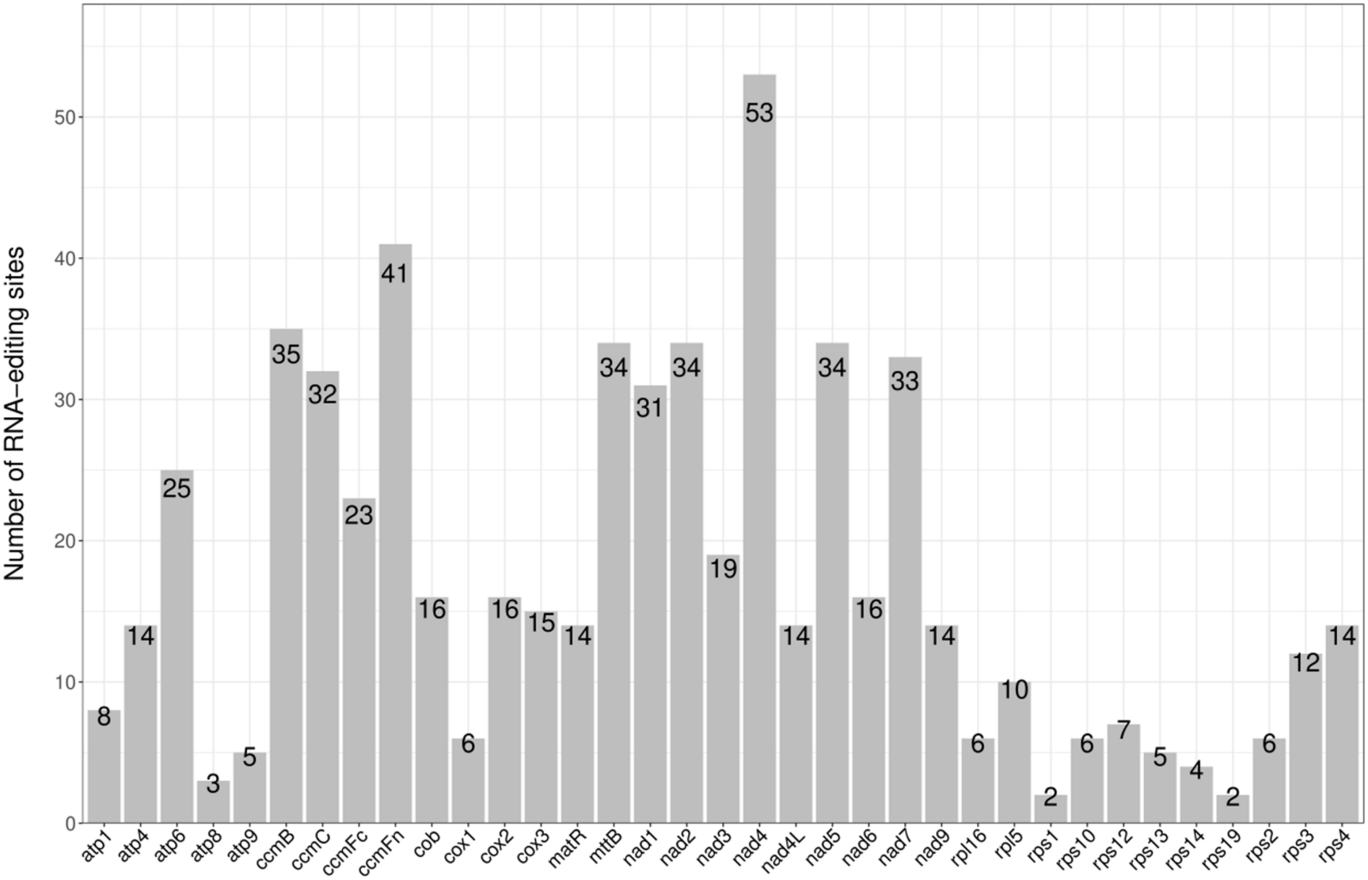
RNA editing sites in protein-coding gene sequence of the mitogenome of *C. wenyujin.* The x-axis displays the gene, and the y-axis displays the number.

RNA editing affects plant growth and development, and in the mitogenome, 506 RNA editing sites were identified from 41 protein-coding genes, all of which were C-U editing sites, and the conversion ratio of second codon position was the largest, with 323, accounting for 63.8% of the total. There were 165 first codon position, accounting for 32.6%, and 18 first and second codon position, accounting for 3.5%. We therefore conclude that the first and second position codon mutations are the main positions where RNA editing induces the amino acid modification, especially in the second position. Among all editing loci, *nad4* and *ccmFn* had the most editing loci, with 53 and 41 respectively. In addition, the data analysis found that NADH dehydrogenase had the most RNA editing sites, with 173, and *nad4* contained the most RNA editing sites of all genes. However, the RNA editing sites encoding maturase gene are the least, only 14, indicating that the distribution of RNA editing sites is very different among different mitochondrial genes. A total of 14 amino acid transformations were found at all RNA editing sites, of which serine (S) was converted to leucine (L), accounting for 112 (22.1%) (**Figure 11**). Subsequently, the most frequent amino acid conversions were observed from proline (P) to leucine (L) and from serine (S) to phenylalanine (F), accounting for 99 (19.6%) and 81 (16.0%) instances, respectively (**Supplementary Table 7**). Furthermore, our analysis of RNA editing revealed that 40.91% of the amino acids maintained their original hydrophobic or hydrophilic properties. Notably, 49.01% of the amino acids underwent a conversion from hydrophilic to hydrophobic characteristics, while 8.70% exhibited the reverse transition from hydrophobic to hydrophilic (**Supplementary Table 8**). Additionally, we identified that 1.38% of the RNA editing sites resulted in the introduction of premature stop codons, potentially leading to truncated protein products.

### 3.5 Phylogenetic analysis

**Figure 12:**
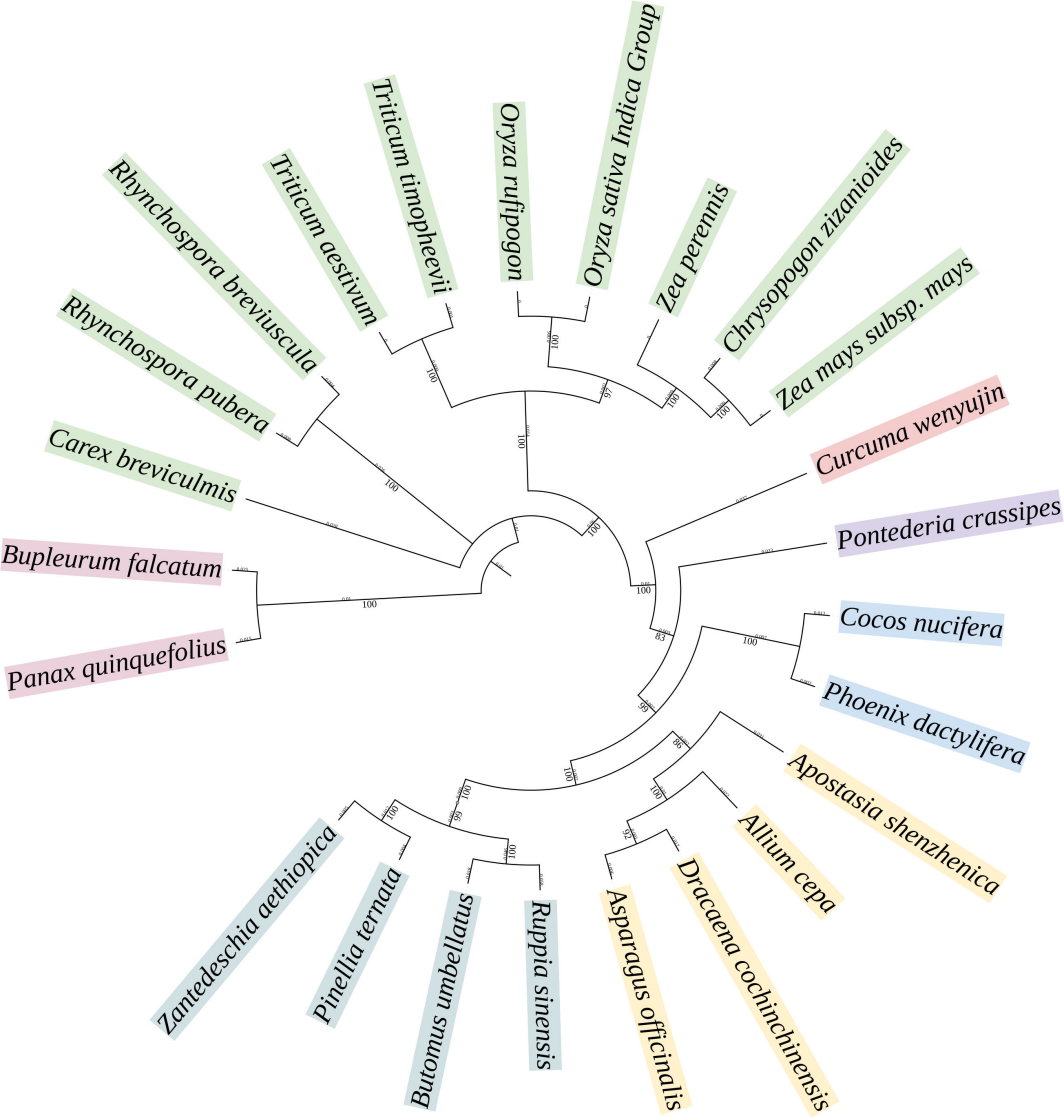
The phylogenetic relationships of *C. wenyujin* with other closely related species. A phylogenetic tree of mitochondrial genome phylogenetic tree was reconstructed based on coding sequences (CDS) from 24 species, with *Panax quinquefolius* and *Bupleurum falcatum* (Apiales) designated as the outgroup.

Given the current lack of publicly available mitochondrial genome data for Zingiberales species, this study constructed a phylogenetic tree based on mitochondrial coding sequences (CDS) encompassing Zingiberales, Poales, Arecales, Asparagales, Commelinales, and Alismatales, with the eudicot order Apiales designated as the outgroup (**Figure 12**; **Supplementary Table 9**). Phylogenetic analysis revealed a close relationship between *C. wenyujin* (Zingiberaceae) and *Pontederia crassipes* (Pontederiaceae, Commelinales). This finding aligns with the APG IV (2016) classification system, which places Zingiberales within the Commelinids clade, and is further supported by shared morphological traits such as pollen morphology and floral architecture. Our results demonstrate that mitochondrial genomic data can serve as a critical molecular resource for elucidating plant phylogenetic relationships, guiding genetic breeding programs, and investigating morphological adaptive evolution.

## 4 Discussion

Medicinal plants hold significant value in healthcare products, medical applications, and economic development (Poole and Penny, 2007; Han et al., 2024). Deciphering the genetic basis underlying these valuable traits is critical for maximizing their utilization. Mitochondrial genomes have attracted increasing attention due to their essential roles in cellular respiration and energy metabolism (Houten and Auwerx, 2004; Hikosaka et al., 2010), as well as in plant adaptation and evolution (Guo et al., 2016; Wu et al., 2024). As an important medicinal species in Zingiberaceae, *C. wenyujin* is an ideal model for genetic research. This study presents the first complete mitochondrial genome of *C. wenyujin*, providing fundamental genomic resources for understanding its genetic basis and evolutionary characteristics.

Previous studies have shown that plant mitochondrial genomes are highly heterogeneous and structurally complex. Most plant mitochondrial genomes range from 200–800 kb (Chen et al., 2022) and exhibit diverse structures including circular, linear, branched, and multipartite forms (Sloan, 2013; Jackman et al., 2020; Jiang et al., 2023). For example, typical circular mitochondrial chromosomes have been reported in *Vitex rotundifolia* and *Bupleurum chinense* (Ma et al., 2022; Wang et al., 2024), while a hybrid linear-circular structure was found in *Mikania cordata* (Bi et al., 2020). Most dicots harbor fewer than five mitochondrial chromosomes, but the monocot *Fritillaria ussuriensis* (Liliaceae) contains 13 distinct chromosomes, all carrying functional genes (Xie et al., 2024). In contrast, the mitochondrial genome of *Manchurian fritillary* comprises 12 circular molecules, with five of them containing only a single functional gene each (Wu et al., 2015). Similarly, 8 of the 27 contigs in the *C. wenyujin* mitochondrial genome lack functional genes. These results indicate substantial structural heterogeneity in both species, with abundant non-coding regions, reflecting the complex architecture of monocot mitochondrial genomes. These findings highlight the high diversity of plant mitochondrial genome organization, including structural variation, size polymorphism, and differential gene distribution (Shen et al., 2019), which can serve as molecular markers for species identification and phylogenetic inference (Kozik et al., 2019; Shen et al., 2019; Wang et al., 2025).

The size variation of plant mitochondrial genomes is mainly caused by sequence insertions, deletions, and the accumulation of repetitive elements. Large repeats (>1 kb) in angiosperms often induce homologous recombination and structural isomerization (Guo et al., 2016; Wynn and Christensen, 2019). In C. wenyujin, we detected long repeats up to 1,727 bp, indicating frequent structural rearrangements. Comparative studies have shown that AT-rich SSRs are abundant in both mitochondrial and chloroplast genomes (Kuang et al., 2011; Qian et al., 2013), likely due to the lower thermodynamic stability of A-T base pairs, which is consistent with our observations.

Comparative analysis of repeat composition between Zingiberales and related orders revealed distinct patterns. The orchid *Dendrobium loddigesii* contains 146 SSRs dominated by AT-rich mononucleotide repeats (Tong et al., 2024), whereas *Camellia japonica* (Theaceae) has 269 SSRs with a high proportion of tetranucleotide repeats (40.15%) (Liang et al., 2025). In this study, we identified 2,171 SSRs in *C. wenyujin*, dominated by tetranucleotide repeats, with 98% showing AT bias and the longest repeat reaching 1,727 bp. These results suggest that SSRs may promote genome fragmentation via repeat-mediated recombination, contributing to the multicircular structure. The high abundance of SSRs may also explain the large size of the *C. wenyujin* mitochondrial genome.

Relative Synonymous Codon Usage (RSCU) reflects deviations from random codon usage (Tang et al., 2022). Codon analysis of *C. wenyujin* mitochondrial PCGs showed three main characteristics: (1) A strong bias toward Leu, Ser, and Arg, with low usage of Trp, consistent with angiosperm mitochondria; (2) Preference for 31 codons, especially AUG, GCU, UAA, CAU, and CAA. Comparative analysis with *Stemona tuberosa* and *Silene noctiflora* (Xu et al., 2025; Zhang et al., 2025) revealed conserved codon usage across distant lineages; (3) A strong A/T bias at the third codon position, as reported in other plants (Romero et al., 2000; Shidhi et al., 2021). This pattern likely reflects long-term adaptive evolution.

RNA editing is a common post-transcriptional modification in plant mitochondria and a major source of genomic variation in angiosperms (Lai et al., 2022). It promotes adaptive evolution by optimizing protein folding (Bi et al., 2016) and improving RNA splicing (Jiang et al., 2023). Studies in dicots such as *Acer truncatum* and *Phaseolus vulgaris* identified 421 and 506 C-to-U editing sites, mainly at the first and second codon positions, consistent with Sloan’s evolutionary model (Sloan et al., 2010; Bi et al., 2020; Ma et al., 2022). In *C. wenyujin*, we detected 506 editing sites across 41 PCGs with similar positional bias. Editing can generate premature stop codons, potentially regulating gene expression. NADH dehydrogenase genes had the highest editing density, while maturase genes had the lowest. RNA editing increased hydrophobicity in 49.01% of amino acid changes, enhancing protein stability, similar to observations in *Dendrobium loddigesii* (Tong et al., 2024). Despite limitations in prediction algorithms and sequencing depth (Edera et al., 2021), our results provide a solid basis for functional genomic studies.

Phylogenetic analysis showed that *C. wenyujin* (Zingiberales) is closely related to *P. crassipes* (Commelinales). This relationship is supported by: (1) Taxonomic affinity: both belong to the Commelinids; (2) Morphological synapomorphies: including parallel venation and specialized floral traits (Givnish et al., 2018); (3) Biogeographic overlap in tropical-subtropical monsoon regions, indicating convergent adaptation (Chase et al., 2016).

## 5 Conclusions

In this study, we sequenced, assembled, and annotated the *C. wenyujin* mitogenome. The total mitogenome length of *C. wenyujin* was 7,856,194 bp and the GC content was 43.91%, showing 27 structures. The genome contains 125 genes, including 41 protein-coding genes, and 66 tRNAs, 9 rRNAs, and 9 pseudogenes. The *C. wenyujin* mitogenome predicted A total of 506 RNA editing sites and exhibited a strong bias toward A/T bases. At the same time, it was found that *C. wenyujin* and *P. crassipes* were closely related through phylogeny. The results of this work will support further research on the mitogenome of *C. wenyujin* in the future.

## Data Availability

The raw data supporting the conclusions of this article will be made available by the authors, without undue reservation.
